# Effects of Flavonoids and Phenols from *Moringa oleifera* Leaf Extracts on Biofilm Processes in *Xanthomonas campestris* pv. *campestris*

**DOI:** 10.3390/plants12071508

**Published:** 2023-03-30

**Authors:** Riccardo Fontana, Anna Caproni, Mariaconcetta Sicurella, Stefano Manfredini, Anna Baldisserotto, Peggy Marconi

**Affiliations:** 1 Department of Chemical, Pharmaceutical and Agricultural Sciences, University of Ferrara, 44121 Ferrara, Italy; 2 Department of Environmental Sciences and Prevention, University of Ferrara, 441211 Ferrara, Italy; 3 Department of Life Sciences and Biotechnology, University of Ferrara, 44121 Ferrara, Italy; 4 Technopole of Ferrara, LTTA Laboratory for Advanced Therapies, Ferrara 44121, Italy

**Keywords:** antimicrobial activity, natural compounds, phytocomplex, scanning electron microscopy

## Abstract

*Xanthomonas campestris* pv. *campestris* is the causal agent of black rot in crucifers, a plant disease with significant economic impact. Xanthomonadaceae is a large family of Gram-negative bacteria that cause symptoms by blocking water flow in plants by invading the xylem. To accomplish this, the main mechanism the bacteria use to adapt to environmental changes and colonize tissues is biofilm formation. In recent years, growing interest in natural antimicrobial compounds has led to the study of different phytocomplexes derived from plants. In this work, *Moringa oleifera* was selected, as its leaves are rich in phenols, essential oils, and vitamins that exert antibacterial activity. *X. campestris* pv. *campestris* biofilm, one of its major virulence factors, was studied. Biofilm formation and removal were analyzed on abiotic and biotic surfaces with and without *M. oleifera* leaf extracts. The data from the analysis show that *Moringa oleifera* leaf extracts and single phenols were able to inhibit biofilm growth on abiotic surfaces, but the activity of the whole phytocomplex was significantly higher compared to that of individual phenols. The effect of *Moringa oleifera* extracts on cabbage leaves in vivo was also found to be very important, as scanning electron microscopy showed that treatment with the extracts led to clear unblocking of the xylem, implying many advantages for use in black rot control.

## 1. Introduction

*Xanthomonas campestris* pv. *campestris* (Xcc) is a Gram-negative bacterium that causes black rot in cruciferous plants by invading the xylem and blocking the water flow in plants [1]. It damages plant cells, which causes tissue maceration and blockage of the xylematic vessels. Biofilms are structural communities consisting of aggregates of bacterial cells protected by a polymeric matrix that facilitates their survival in many environments [2]. Once it reaches the xylem, *X. campestris* pv. *campestris* colonizes it due to the action of pili and the production of extracellular polysaccharides (EPS), such as xanthan gum, as well as cellulase, protease, polygalacturonase, amylase, hemolysin, and hemagglutinin [3,4]. Furthermore, the type III secretion system participates in the pathogenicity mechanism, as it can interfere with plant defense apparatuses [5].

Black rot is one of the most destructive diseases in the world, with a significant economic impact in many geographic regions [6]. Chemical approaches have been widely used to control *X. campestris* pv. *campestris*, but these have led to environmental pollution and the emergence of resistant strains [7,8,9,10]. In particular, copper and streptomycin resistance has been observed in many pathogenic bacteria; copper mixtures are not always able to efficiently control disease, which leads to the selection of resistant bacterial isolates. Furthermore, copper-based compounds can interrupt the microbial activity in soil, as it adversely affects the activity of microorganisms and earthworms, leading to slower decomposition of organic matter [11,12].

In recent years, growing interest in natural antimicrobial compounds that may be bio-safe and eco-friendly have led to the study of different phytocomplexes derived from plants [13]. In this work, *Moringa oleifera* Lam. (MOL) was selected, which has leaves known to be rich in phenols, flavonols, essential oils, and vitamins that exert antibacterial activity [14]. Previously, we reported high concentrations of flavonols and flavones in *M. oleifera* Lam. leaves [15,16]; several glycosylated flavonoids have also been identified, including quercetin, kaempferol, and rutin. In the dried leaves, high concentrations of phenolic acids have been detected, such as chlorogenic acid, gallic acid, ferulic acid, ellagic acid, and caffeic acid [14,16,17]. Among them, chlorogenic acid is known to significantly increase membrane permeability in different bacterial pathogens, resulting in the loss of the barrier function, which induces leakage of nucleotide [18].

Previous studies by our group highlighted the effects of antimicrobial activity of different *Moringa oleifera* extracts on phytopathogens [15,16]. The main components of the phytocomplex detected in our extracts were tested individually in different assays in order to better understand the mechanism of action of each flavonoid and phenol in *X. campestris* pv. *campestris* compared to that of the whole phytocomplex. Furthermore, as stated before, biofilm formation is a crucial step in this disease. Biofilms are often studied with scanning electron microscopy, and *X. campestris* pv. *campestris* biofilm formation and removal were analyzed on abiotic and biotic surfaces with and without the influence of *M. oleifera* leaf extracts: *M. oleifera* hydroalcoholic extract (HA-MOL), *M. oleifera* methanolic extract (MeOH-MOL), and *M. oleifera* hydroalcoholic extract with the addition of maltodextrins (HAMD-MOL).

The objectives of this work were to study the phytocomplex and how each individual molecule acts, and to investigate biofilm-associated processes and how they are influenced by the application of *M. oleifera* extracts.

## 2. Results

The potential antimicrobial effects of several phytochemical phenolic compounds on membrane permeability, biofilm formation, and swarming motility of *X. campestris* pv. *campestris* were evaluated: chlorogenic acid (CA), epigallocatechin (EC), rutin (RU), and quercetin (QU) were selected, as these compounds were found to be the most abundant in the studied extracts from *M. oleifera* leaves [15,19,20,21]. One of the main results of this study is the discovery of the antimicrobial potential of *M. oleifera* extracts on *X. campestris* pv. *campestris*. The extract effect was retained in all tested strains, without any significant differences, giving us important information on the use of natural phytocomplexes on Xanthomonadaceae. Despite the genetic differences that may exist between bacterial strains, the extract is able to inhibit bacterial growth and alter membrane permeability. Based on analyses of other phytopathogens, this effect is probably extendable to many Gram-negative bacteria.

### 2.1. Effects of Single Phenols on Membrane Permeability

**Figure 1:**
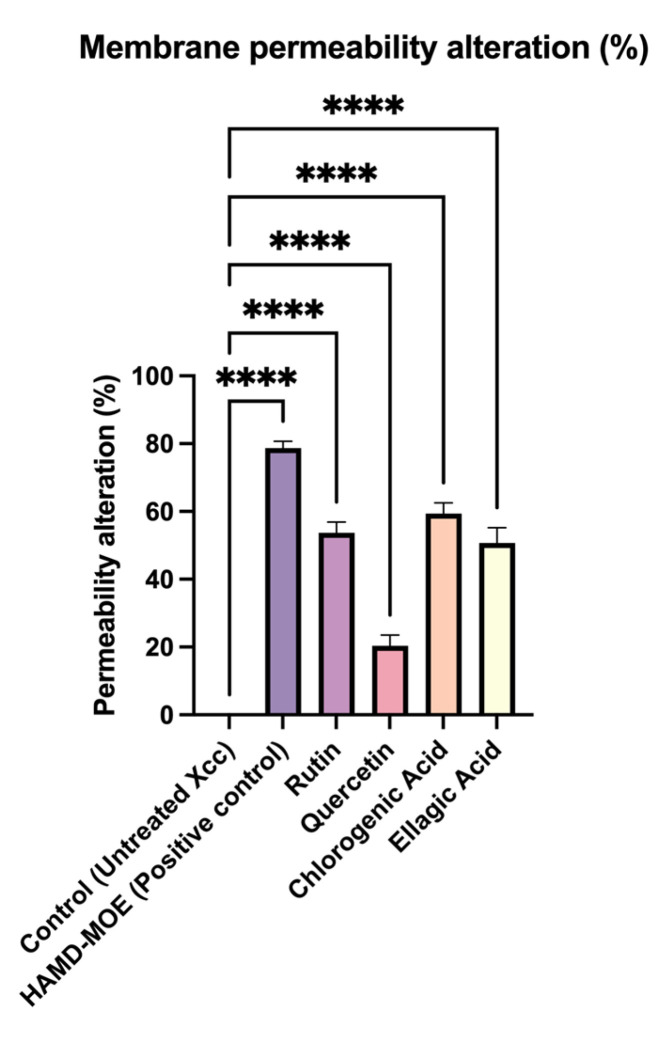
In vitro membrane permeability assay. *X. campestris* pv. *campestris* permeability was assessed by PI intake compared to untreated control. Data represent average of three independent experiments on three copies (mean ± standard deviation), and values are given as percentages; **** *p* < 0.001. Complete statistical analysis can be found in Supplementary Table S3. The analysis performed on the activity of single strains can be found in Supplementary Figure S1.

To evaluate membrane permeability, sub-minimal inhibitory concentrations (MICs) of selected compounds were tested on *X. campestris* pv. *campestris* bacterial suspensions for 60 min. To demonstrate possible membrane weathering, a fluorescent intercalating agent, propidium iodide (PI), was added to the bacterial suspensions. The assay showed that the single compounds provided membrane permeability similar to that of the positive control, represented by the bacteria treated with the most effective extract, the hydroalcoholic extract of *M. oleifera* with maltodextrin (HAMD-MOL) (Figure 1). It was noted that the effects of the phytocomplex were retained and remained higher than those of the single compounds tested (rutin, quercetin, chlorogenic acid, and ellagic acid). However, the experiment also showed that PI could rapidly enter *X. campestris* pv. *campestris* cells after chlorogenic acid, ellagic acid, and rutin were added, while the cell membrane was still polarized [18].

### 2.2. Effects of Single Phenols on Biofilm Formation

**Figure 2:**
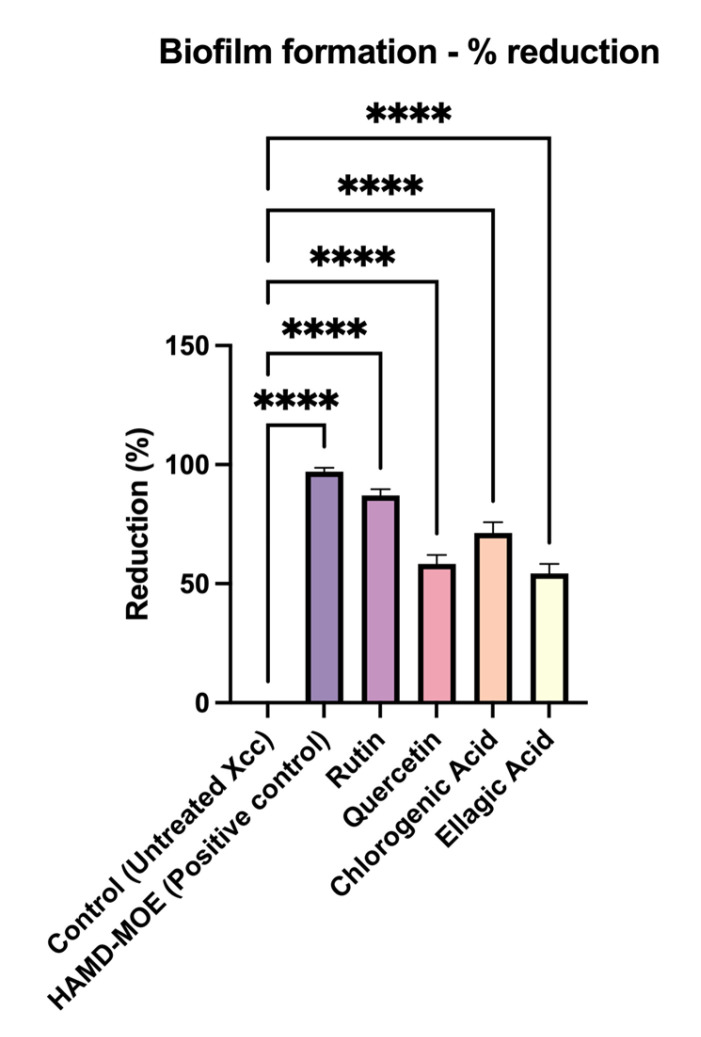
In vitro biofilm formation assay. *X. campestris* pv. *campestris* biofilm formation was assessed with the crystal violet method, presented as a percentage compared to untreated control. Data represent the average of three independent experiments on three copies (mean ± standard deviation), and values are given as percentages; **** *p* < 0.001. The analysis performed on the activity of single strains can be found in Supplementary Figure S2.

To evaluate whether each isolated compound had anti-biofilm activity, sub-MICs were added to the bacterial suspension. After incubation, biofilm formation was quantified using a spectrophotometer. As shown in Figure 2, compared to the positive control, consisting of only overnight bacterial suspension of *X. campestris* pv. *campestris*, there was a marked reduction in biofilm formation for all compounds tested. In this case, rutin seemed to be the most effective, reducing biofilm formation by 91%, compared to the control, followed by chlorogenic acid (70% reduction).

### 2.3. Assay of Biofilm Formation and Removal on Abiotic Surfaces

**Figure 3:**
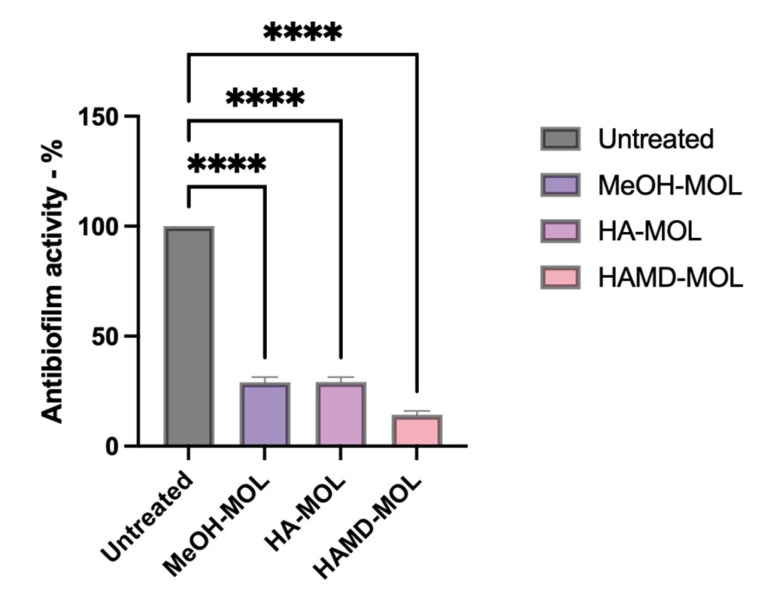
In vitro biofilm formation assay. *X. campestris* pv. *campestris* biofilm was measured by OD_600_, presented as a percentage compared to untreated control. Data represent the average of three independent experiments on three copies (mean ± standard deviation), and values are given as percentages; **** *p* < 0.001. The analysis performed on the activity of single strains can be found in Supplementary Figure S3 and Table S4.

**Figure 4:**
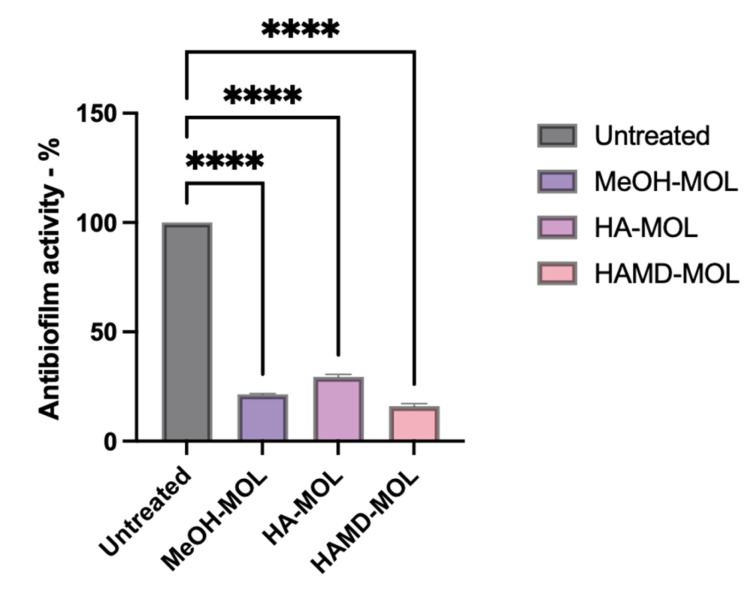
In vitro biofilm removal assay. *X. campestris* pv. *campestris* biofilm was measured by OD_600_, presented as a percentage compared to untreated control. Data represent the average of three independent experiments on three copies (mean ± standard deviation), and values are given as percentages; **** *p* < 0.001. The analysis performed on the activity of single strains can be found in Supplementary Figure S4 and Table S5.

To assess whether *M. oleifera* extracts had anti-biofilm activity, and to visualize which part of the biofilm formation process was affected, biofilm formation on abiotic surfaces (such as a plastic cell strainer) was studied. From the analysis it was possible to confirm the antimicrobial and antibiofilm activity of the alcoholic extracts of *M. oleifera*. In vitro experiments have shown how extracts can inhibit both the formation and removal of biofilm on inert surfaces. As for the formation assay, MeOH-MOL, HA-MOL, and HAMD-MOL were able to reduce biofilm formation by 71, 70.78, and 85.67%, respectively (Figure 3). Similar activity was detected by the removal assay, with removal of mature biofilm by 78.56, 70.12, and 84%, respectively (Figure 4).

As known from our previous analysis, the three extracts contained comparable quantities of flavonoids and total phenols; in fact, their activity did not differ statistically from one another (Supplementary Tables S1 and S2) [16,22].

**Figure 5:**
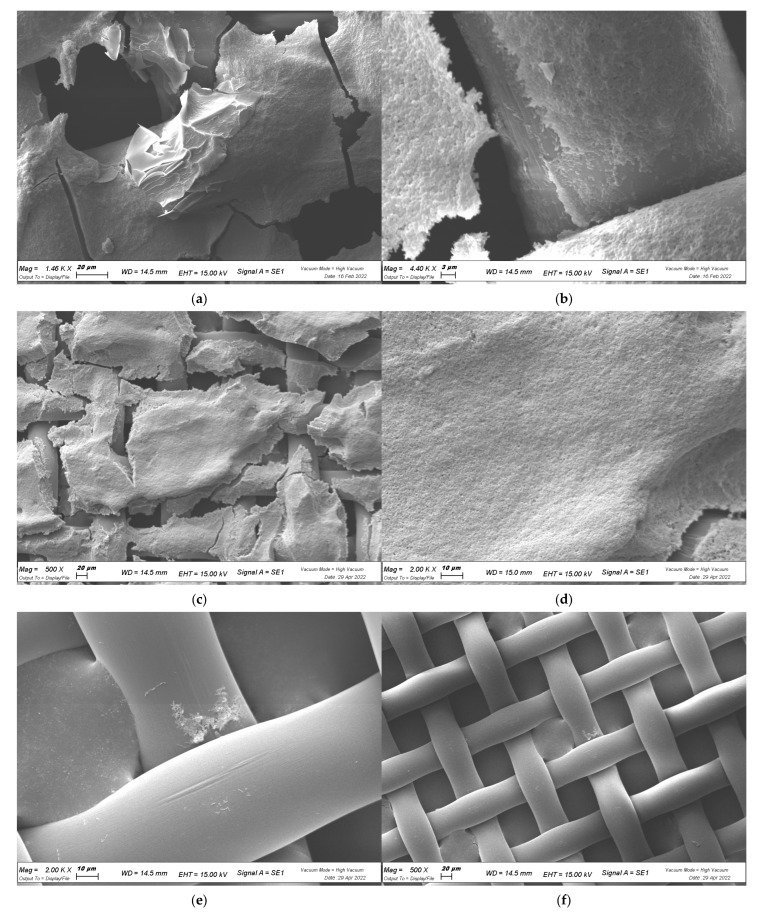

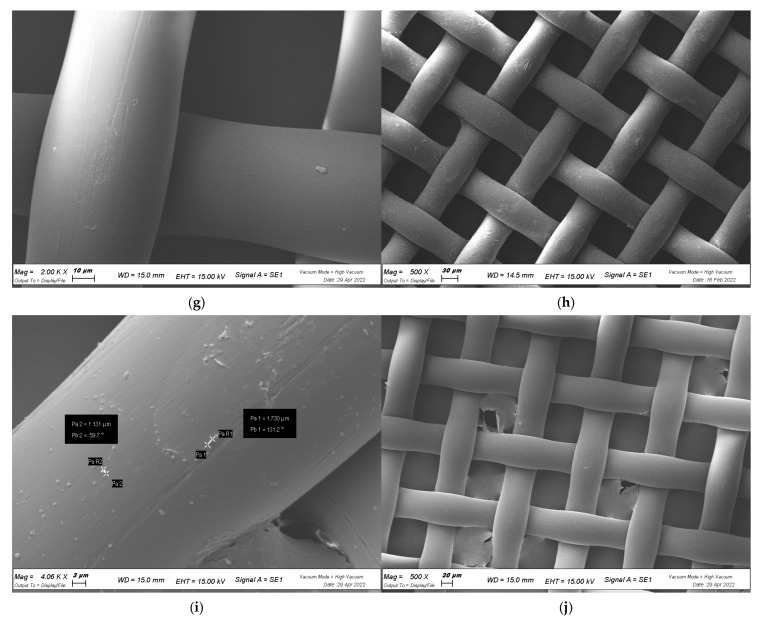
Effects of biofilm formation on abiotic surface by SEM visualization. (**a**–**d**) Control (CTRL) samples; (**e**,**f**) HA-MOL; (**g**,**h**) HAMD-MOL; (**i**,**j**) MeOH-MOL. Controls are shown at magnification of 1.46 K, 4.4 K, 500, and 2 K×. Treated samples are shown at magnification of 2 K and 500×, except for (**i**), which, in order to visualize bacterial size, is shown at magnification of 4.06 K×.

Biofilm growth on a plastic cell strainer was also analyzed with scanning electronic microscopy (SEM). This analysis showed that the bacteria were in distress when in contact with the extracts. Compared to the control, the size of planktonic cells was reduced by half (5 µm for untreated vs. ≤2 µm for treated *X. campestris* pv. *campestris*), with signs of cell suffering such as folding (Figure 5). Focusing on the three-dimensional development of the biofilm, it was evident that untreated cells (Figure 5a–d) had a clearly defined structure, with abundant and very thick cellular clusters characterized by multilayer distribution. An extracellular matrix was abundantly produced during the maturation of the biofilm, and xanthan gum foils could be observed, as shown in Figure 5a. In addition, thin fibers connecting and covering cells were visualized. Interestingly, *X. campestris* pv. *campestris* cells were found in localized groups in the spaces of the cell strainer, where they multiplied to fill the space; the bacteria appeared to be suspended in the air spaces, but they were likely protected by an EPS matrix that dried during sample fixation.

Focusing on the *M. oleifera* extract treated samples, it was clear that bacteria were not able to produce typical biofilm structures (Figure 5e–j). The bacteria were mainly found to be isolated and in their planktonic state; however, it should be noted that *X. campestris* pv. *campestris* started to fill the spaces of the cell strainer even under the effects of MeOH-MOL and HA-MOL, but was not able to cover, adhere to, and colonize the single filaments (Figure 5e,f,i,j). Meanwhile, HAMD-MOL was shown to have a better effect, as it completely inhibited the release of EPS (Figure 5g,h). No extracts allowed the formation of a multilayer biofilm, indicating that quorum sensing (QS) might have been inhibited [23].

### 2.4. In Planta Biofilm Formation

**Figure 6:**
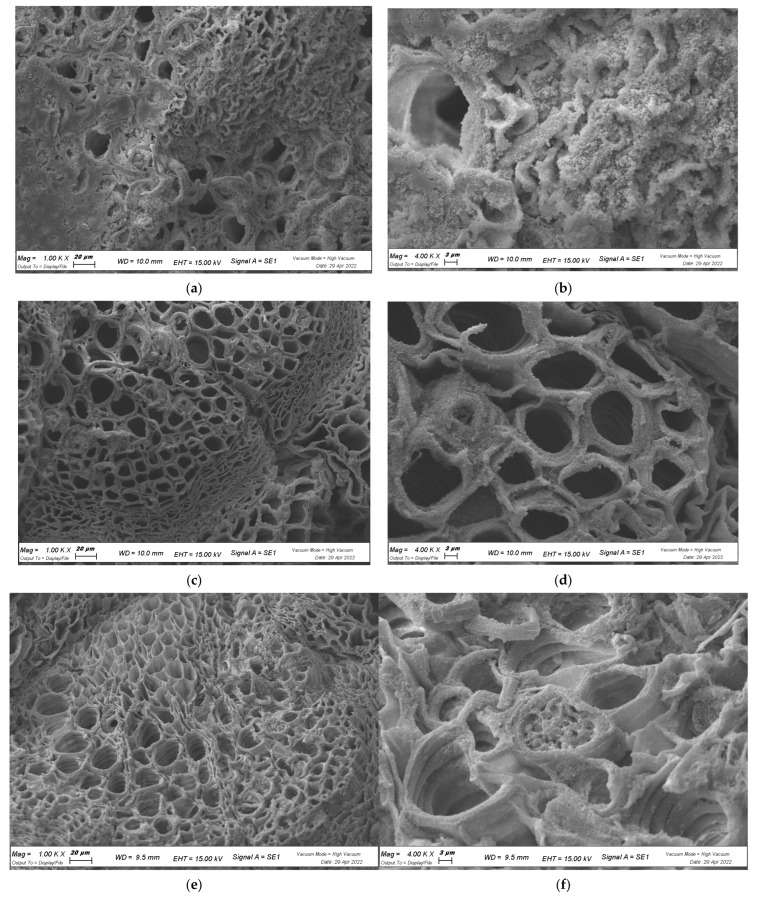

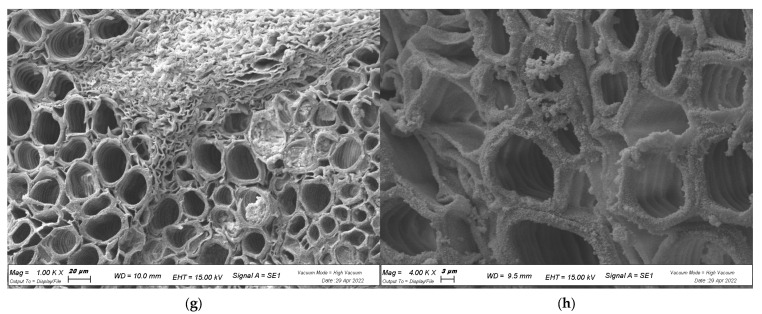
Effects of biofilm formation on biotic surface, SEM visualization: (**a**,**b**) CTRL samples; (**c**,**d**) HAMD-MOL; (**e**,**f**) HA-MOL; (**g**,**h**); MeOH-MOL. All images are shown at 1 K and 4 K× magnification.

To evaluate whether extracts blocked biofilm formation in planta, cabbage leaves, as natural hosts of *X. campestris* pv. *campestris*, were used. Colonization of the biotic surface is shown in Figure 6. Cabbage leaves were inoculated with a suspension of *X. campestris* pv. *campestris*, followed by *M. oleifera* extract injection and spraying. The progression of infection was visually monitored at different time points. After the infection was clearly established, cabbages were sliced and visualized with SEM. In the untreated inoculated cabbage, the vessels were completely coated by bacteria and EPS, mainly localized and attached to the inner surface of vessels (Figure 6a,b). When the extracts were applied, the quantity of living bacteria was significantly reduced and localized in a few vessels, with only a few tridimensional structures formed, suggesting that the extracts might not have been able to reach all parts of the cabbage leaf (Figure 6c–h). Again, HAMD-MOL (Figure 6c,d) had the strongest effect in terms of inhibiting bacterial adhesion and growth through the vessel. This effect was also confirmed by the total viable bacteria count from the cabbage leaf core (1 × 1 cm^2^): the assay showed that untreated cabbage leaves were completely invaded by *X. campestris* pv. *campestris*, corresponding to a growth of 2.6 × 10^8^ CFU/mL, while treated leaves showed an average 4- to 5-fold log reduction in bacterial growth, corresponding to 3.03 × 10^4^, 2.59 × 10^4^, and 6.02 × 10^3^ CFU/mL for MeOH-MOL, HA-MOL, and HAMD-MOL, respectively, as shown in Table 1.

In conclusion, SEM images showed complete colonization by *X. campestris* pv. *campestris* on abiotic and biotic surfaces when not under the effects of extracts; in fact, the control showed that the fibers of the cell strainer were coated with EPS, and cabbage xylems were completely invaded. Furthermore, the production of xanthan gum (one of the main components of *X. campestris* pv. *campestris* EPS and virulence factors) layers was observed. The antibiofilm properties of HAMD-MOL and MeOH-MOL were proven and confirmed by the presence of just a few solitary planktonic bacteria and no observed complex or organized structures.

## 3. Discussion

Global research on bacterial biofilms continues on many fronts, with particular emphasis on specifically expressed genes and the role of biofilm in acquiring resistance, as well as the evaluation of control measures and the development of innovative strategies [24,25].

It is known that many natural bioactive molecules have antibiofilm (as well as antimicrobial) activity. This is particularly important in the case of xylematic pathogens; thus, biofilm formation during *X. campestris* pv. *campestris* growth was investigated, as it plays a key role in the infection process. The extracts used in this study showed significant antibacterial activity against *X. campestris* pv. *campestris* in the biofilm matrix, apparently limiting the adhesion of bacteria to the surface. The extracts also displayed effective biofilm-dissolving ability, supporting their possible use in therapeutic treatments, and the activity could not be completely expressed by the individual molecules detected in MOL leaves. These findings support the assumption that phenolic compounds such as quercetin, rutin, ellagic acid, and others can act cooperatively as antibiofilm compounds, as none of the individual phenols reached the same activity as that of the whole phytocomplex [26].

One of the main differences found was the higher activity of the HAMD-MOL extract over time. The activity was more pronounced when maltodextrins were added, as the tested extracts are comparable in terms of polyphenol percentage. The maltodextrin (MD) hydroalcoholic extract had the highest effect because maltodextrins act as coating agents integrating bioactive molecules, which extends their lifespan and prevents them from losing their activity. A study by Sri Harsha et al., found that the addition of maltodextrins significantly enhanced the stability of certain polyphenolic compounds, particularly chlorogenic acid [22]. Recently, a study found that combined treatment with electrochemically generated H_2_O_2_ and maltodextrin was more effective at reducing the density of viable biofilm cells than any single treatment, and another study showed that gum arabic and maltodextrin microencapsulation resulted in significant preservation of pepper seed oil against oxidation during storage [27,28,29].

Based on our previous studies and a literature review, we know that the phytocomplex consists of polyphenolic molecules that can alter the permeability of the bacterial membrane, leading to a shutdown of ATP synthesis, causing the cessation of all ATP-dependent functions and a subsequent reduction in selectivity of compounds that can penetrate the bacterial cytoplasm [14,30,31]. In fact, some of these molecules act by modifying membrane permeability and integrity by promoting the passage through the cytoplasm of compounds capable of inhibiting the enzymatic complexes involved in bacterial replication [32].

Specifically, among the simple phenols, flavonoids, and phenolic acids detected in the leaves of *M. oleifera* Lam., naringenin, ferulic acid, rutin, chlorogenic acid, and ellagic acid may complex with the bacterial cell walls, affecting the fluidity, structure, and function of the phospholipid double layer. In particular, the antibacterial activity of rutin and quercetin has been linked to their solubility and interaction with the bacterial cell membrane, which is largely determined by quercetin’s hydroxyl groups [33,34,35]. Recent studies have shown that flavonoids (quercetin in particular) can effectively disrupt the integrity of the bacterial membrane, resulting in inhibition of bacterial growth [26,32,36]. Using TEM analysis, Wang et al., observed that in treated *E. coli*, there were numerous structural anomalies in damaged cells: wall lysis, deformation, leakage of cellular material, and unequal endochyleme density. Eventually, cell cavitation and cell death were evident. Similarly, in treated *X. campestris* pv. *campestris*, membrane shrinking and cavitation were noted [18,36]. In a recent study, flavonoid activity was investigated, comparing ceftriaxone and imipenem, since both are bacterial cell wall inhibitors. Alnour et al., suggested that the tested antibiotics caused the cell wall damage, while lesions in the cytoplasmic membrane were inflicted by flavonoids, with greater damage observed when a combination of flavonoids was used [37].

Some molecules, such as robinetin, myricetine, and epigallocatechin, act by creating hydrogen bonds with nucleic bases, inhibiting both DNA and RNA synthesis. Flavonoids with a hydroxyl central ring, such as rutin, bind to the β-subunit of DNA gyrase (GyrB) and block the binding site for ATP, thereby compromising processes such as cell division and chromosome replication, leading to inhibition of bacterial growth, as observed by Plaper et al. [38]. According to Liu et al., quercetin inhibited the supercoiling activity of gyrase in *Lactobacillus* spp., ultimately leading to the disruption of bacterial DNA replication [39,40]. In addition, rutin is able to inhibit the type II topoisomerase enzyme, promoting DNA cleavage, while myricetin appears to inhibit a number of enzymes of fundamental importance, such as dihydrofolate reductase and different DNA and RNA polymerases [2,41]. In this work, the experiments on membrane permeability showed that PI could rapidly enter *X. campestris* pv. *campestris* cells after chlorogenic acid was added, whereas the cell membrane was still polarized, in accordance with a study by Lou at al. [18]. These results suggest that chlorogenic acid might first disrupt the bacterial membrane permeability, and subsequently depolarize it. This condition then leads to *X. campestris* pv. *campestris* being exposed to other molecules in the complex, resulting in bacterial cell death.

The formation of biofilms is a crucial mechanism in the pathogenesis of black rot on crucifers. Biofilms allow bacterial colonies to express resistance in a homogeneous and cohesive way, through improved inter-cell communication in stressful environmental conditions [42,43,44,45]. Flavonoids, especially quercetin, are also able to interfere in the pathways involved in quorum sensing, thereby preventing bacterial adhesion and biofilm formation. A recent study determined that quercetin interferes with the functioning of proteins involved in glycolytic pathways, protein folding, and protein elongation, while Manner et al., reported that quercetin inhibited the production of violacein pigment, an indicator of bacterial QS in *Chromobacterium violaceum* [46,47].

Focusing on phytopathogens, our most recent work showed how the extracts inhibit the production of amylovoran (one of the main virulence factors of *Erwinia amylovora*, involved in QS mechanisms) in *Erwinia amylovora* cells, confirming its activity in the inhibition of QS signaling [15]. The modifying of membrane integrity and permeability and the action of flavonoids on GyrB result in a considerable dissipation of energy. Therefore, this membrane condition has implications for a variety of ATP-dependent mechanisms, such as motility. In fact, an analysis carried out in our recent work showed that *X. campestris* pv. *campestris* treated with *M. oleifera* extracts, though subjected to energy shortages, was not able to swarm on soft agar plates [15,16]. In the previous analysis, *X. campestris* pv. *campestris* was unable to completely attach to either an abiotic or biotic surface and form a mature biofilm.

The dispersal of bacteria into plant vessels is a process finely regulated by a detection system that perceives and records the characteristics of the surrounding environment, indicating the direction toward a more favorable environment conducive to survival [48,49,50,51]. In this sense, the bacteria try to limit their movement as much as possible to reduce contact with the extracts. Focusing on the cabbage infection, we observed that *X. campestris* pv. *campestris* did not move freely along the xylem, but created small masses of bacterial cells that were not able to form a complete biofilm and start the infection. The reduced effectiveness of ATP-dependent mechanisms also means a slowing down, if not a complete cessation, of the processes involved in the secretion of molecules necessary for cell-to-cell communication and, finally, biofilm formation. This may explain the analyzed antibiofilm effect of *M. oleifera* Lam. extracts on *X. campestris* pv. *campestris* isolates; in fact, the ability of some catechins and flavonols to effectively counteract cell adhesion, the initial stage of biofilm formation, was found in *E. coli* and *S. aureus* [52]. Ellagic acid, tannic acid, and epigallocatechin gallate, on the other hand, appear to inhibit biofilm maturation, an effect likely associated with wall damage from peptidoglycan cleavage [53,54,55].

As the anti-biofilm activity of the extracts has been defined, more in-depth studies will be needed to evaluate the stage of biofilm formation that is most compromised.

## 4. Materials and Methods

### 4.1. Bacterial Strains and Media

The *X. campestris* pv. *campestris* isolates used in this study were given by the Emilia Romagna Plant Health Agency (Servizio Fitosanitario Regione Emilia-Romagna), and strain 3586 (ATCC 33913) was used as a control. Strains were isolated from brassica seeds in Bologna, Ravenna, and Forlì-Cesena provinces, as listed in Table 2. Bacterial strains were conserved at −80 °C in Luria–Bertani (LB) broth (Liofilchem, Roseto degli Abruzzi, Italy) with 50% glycerol. During the study, bacteria were cultured in LB broth [56] and plated on R2A agar (18.1 g/L; Scharlab, Lodi, Italy) or LB agar (30 g/L; Liofilchem) and incubated at 25 and 28 °C.

The bacteria listed in Table 2 were isolated from different plant samples in different parts of the region. The tests were run with all strains listed in the table, but no statistical differences in the response to the extracts were detected between strains; therefore, all results were used and are reported as a mean. The analysis performed on the activity of all single strains can be found in Supplementary Materials.

### 4.2. Plant Material and Extraction Methods

Leaves of *M. oleifera* were collected in August 2019, and were subsequently dried by Evra S.r.l. (Loc. Galdo, Lauria, Italy). Dried samples were packaged and sent to the laboratory. Upon arrival, dried leaves were ground to a fine powder with a mortar and stored at −80 °C. The extraction process was carried out by adapting a previously described method briefly discussed below [13]. The extracts were stored at −18 °C until use. Quantification results can be found in Supplementary Tables S1 and S2.

The extracts were formulated as follows:

- Hydroalcoholic extract (HA-MOL): 10 g of powder was mixed with 200 mL of hydroalcoholic solution (ethanol:water, 70:30) at room temperature for 1 h under magnetic stirring, then filtered and concentrated in vacuum to provide the desired dry hydroalcoholic extract.
- Methanolic extract (MeOH-MOL): 5 g of powder was mixed with 100 mL of methanol and subjected to two sonication cycles (40 °C, 60 min, 80%) with subsequent centrifugation. The supernatant was concentrated under vacuum to obtain the desired product.
- Hydroalcoholic extract with maltodextrin (HAMD-MOL): dried *M. oleifera* Lam. leaves were extracted with 50% ethanol (raw material:solvent, 1:10) for 45 min at 45 °C. After filtration, concentration, and pasteurization, the extract was spray-dried using maltodextrin, obtaining a fine powder.

### 4.3. Effects of Single Phenols on Membrane Permeability

The effects of individual phenols on membrane permeability were assessed with propidium iodine (PI) staining. PI does not penetrate membranes under normal circumstances, but when the integrity and permeability of the structure changes, it enters bacterial cells and intercalates between DNA bases; this intercalation can be read with a spectrofluorometer, allowing differentiation between healthy and functional cells within damaged cells that have a non-functional membrane structure [57,58]. The experiment was conducted as previously established [16]. Bacterial suspensions were grown overnight in Luria Broth (LB) for 24 h at 25 °C. After incubation, 1 × 10^5^ CFU/mL of bacteria was placed in 4 sterile Eppendorf tubes containing *M. oleifera* Lam. HAMD extract at concentrations corresponding to 2× MIC (0.2 mg/mL), 1× MIC (0.1 mg/mL), 1/2× MIC (0.05 mg/mL), and 1/4× MIC (0.025 mg/mL), as previously described [16], with sub-MICs of different phenols (chlorogenic acid, 10 µg/mL; rutin, 100 µg/mL; quercetin, 100 µg/mL; ellagic acid, 250 µg/mL). Rutin (Sigma Aldrich, St. Louis, MO, USA, CAS 207671-50-9), quercetin (Sigma Aldrich CAS 117-39-5), and ellagic acid (Sigma Aldrich CAS 476-66.4) were suspended in sterile water, while chlorogenic acid (Sigma Aldrich CAS 327-97-9) was dissolved in 1× PBS. The procedure was repeated by varying the incubation time, so the bacterial suspensions containing different concentrations of single phenols were incubated for 180, 120, 60, and 5 min in a static incubator at 25 °C. After incubation, the suspensions were centrifuged for 5 min at 14,000 rpm and washed with 1× PBS (137 mM NaCl, 2,7 mM KCl, 8 mM Na_2_HPO_4_, 2 mM KH_2_PO_4_). The pellet was then resuspended with propidium iodide (0.5%) and incubated for 15 min, avoiding exposing the suspension to the light. Then, the suspensions were plated on 96-well plates, and the values were read through a fluorescence microplate reader (Tecan-Fluoroscan, Tecan Italia, Cernusco sul Naviglio, Italy) [57]. All analyses were performed using data obtained from 3 different experiments in triplicate.

### 4.4. Effects of Single Phenols on Biofilm Formation

The effects on biofilm formation were determined by the crystal violet method, as described by Wilson et al. [59]. According to the cited protocol, higher concentrations of bacteria are needed to perform biofilm assays. *X. campestris* pv. *campestris* suspensions containing 10^6^ CFU/mL were inoculated in Luria Broth with *M. oleifera* extracts and single phenols at non-lethal concentrations in a 96-well U-bottom microplate for 72 h at 25 °C. The non-lethal concentrations of single phenols were as follows: chlorogenic acid, 10 µg/mL; rutin, 100 µg/mL; quercetin, 100 µg/mL; and ellagic acid, 250 µg/mL. After incubation, the growth media, extracts, phenols, and planktonic cells were removed from the plate and washed with sterile deionized water. Crystal violet 1% was added to each well and incubated for 30 min at room temperature, and the dye solution was washed off the plate several times with deionized water. Then 200 μL of decoloring solution (90% ethanol) was added to each well and incubated for 15 min at room temperature to increase crystal violet solubility. The contents of the 96-well plate were then transferred to a new clean microplate and biofilm formation was quantified by reading the absorbance at 570 nm through a microplate reader (Tecan-Sunrise, Tecan Italia, Cernusco sul Naviglio, MI, Italy). All analyses were performed using data obtained from 3 different experiments in triplicate.

In order to obtain information on reduced bacterial biofilm after exposure to *M. oleifera* extracts, bacterial colonization and biofilm matrix synthesis were examined by biofilm formation assays. Biofilm formation was tested on an abiotic surface, cell strainer fibers, and a biotic surface, cabbage leaves, a natural host of *X. campestris* pv. *campestris*.

### 4.5. Assay of Biofilm Formation and Removal on Abiotic Surface

Bacterial adhesion to an inert surface was assayed in a 6-well polypropylene plate. *X. campestris* pv. *campestris* suspensions containing 10^6^ CFU/mL were inoculated in LB in a 6-well plate with a cell strainer insert (BD Italia, Milano, Italy). For biofilm formation assay, MOEs were added along with the bacteria; for biofilm removal assay, MOEs were added after 72 h.

For the biofilm formation assay, bacteria (10^6^ CFU/mL) were incubated for 72 h in Luria Broth medium with *M. oleifera* extract at non-lethal concentrations. The medium was changed every 24 h, and then tested with crystal violet (CV) staining.

For the biofilm removal assay, *X. campestris* pv. *campestris* suspensions containing 10^6^ CFU/mL were inoculated in a cell-strainer insert for 72 h and then treated with the extracts for 24 h. Biofilms, generated as described above, were removed and processed by crystal violet staining.

CV 1% was added to each well and incubated for 30 min at room temperature [59,60]. The dye solution was removed by washing the plates 3 times with deionized water for biofilm formation assay, and until a clear solution was obtained for biofilm removal. Then 200 µL of decoloring solution (90% ethanol) was added to each well and incubated for 15 min at room temperature to increase CV solubility. The contents of the 6-well plate were then transferred to a new clean 96-well microplate, and the biofilm was quantified by reading the absorbance at 570 nm through a microplate reader (Tecan-Sunrise, Tecan Italia, Cernusco sul Naviglio, Italy). All analyses were performed using data obtained from 3 different experiments in triplicate.

### 4.6. Biofilm Formation in Planta and Total Viable Count

*X. campestris* pv. *campestris* strains were grown overnight in 5 mL of LB broth at 25 °C for 24 h, centrifuged, and then resuspended with 1× PBS to reach a bacterial concentration of 5 × 10^7^ CFU/mL. In order to colonize plant tissues, bacteria must be highly concentrated with respect to in vitro experiments [61]. Cabbage leaves were superficially sterilized with a 0.7% solution of sodium hypochlorite and then washed with sterile water, delicately perforated with a sterile 10 µL tip, and inoculated with 5 µL of the bacterial suspension. After the drop was absorbed within 30 min, the MIC of each active extract (HA-MOL, 0.5 mg/mL; MeOH-MOL, 0.5 mg/mL; HAMD-MOL, 0.1 mg/mL) was inoculated in the same hole. The cabbage leaves were then incubated at room temperature (ranging from 20 to 22 °C) for 6 days.

After the incubation time, infected and treated cabbage leaves were cut into small pieces, corresponding to an area of 1 × 1 cm^2^, and soaked in 10 mL of 1× PBS for 1 h. Then 1 mL of PBS was diluted up to 10^−9^, plated on R2A agar plates, and incubated for 48 h at 25 °C. After incubation, colonies were counted to record bacterial cell viability.

In addition, the leaves were cut in order to record the area of necrosis around the inoculation point and treated for scanning electron microscopy (SEM) observation as described below.

### 4.7. Visualization of Biofilm with SEM

Ultrastructural analysis was performed by SEM to reveal the cell interaction traits. *X. campestris* pv. *campestris* was grown on cell strainers for 72 h and then fixed in 2.5% glutaraldehyde in 0.1 M KPO_4_ buffer. After being washed with sterile distilled water (SDW), samples were dehydrated by increasing concentrations of ethanol and dried with a critical point dryer. The samples were mounted on metal stubs and coated in gold/palladium. Samples were examined with a Zeiss Evo40 SEM with an electron high tension (EHT) accelerating voltage of 15.00 KV [62].

Visualization of *X. campestris* pv. *campestris* on biological surfaces was also achieved by SEM. Previously sterilized cabbage leaves were inoculated with *X. campestris* pv. *campestris* as described above and treated with the extracts (HA-MOL, 0.5 mg/mL; MeOH-MOL, 0.5 mg/mL; HAMD-MOL, 0.1 mg/mL). Pieces of inoculated leaves (<6 mm) were fixed in 3% glutaraldehyde in 0.1 M KPO_4_ buffer. After being washed with SDW, samples were dehydrated by increasing concentrations of ethanol and dried with a critical point dryer. The samples were prepared and observed as described above.

### 4.8. Statistical Analysis

All tests were performed 3 times in triplicate, and statistical analysis was performed using one-way ANOVA followed by Dunnett’s multiple comparisons test with GraphPad Prism version 9.0.0 for MacOS (GraphPad Software, San Diego, CA, USA, www.graphpad.com; accessed on 8 January 2021), with *p* ≤ 0.05 indicating significant differences. The detailed statistical analysis is available in the Supplementary Materials, accompanied by a detailed extraction and chemical quantification methodology, as performed, and described, in previous works [63,64,65,66,67].

## 5. Conclusions

The problematic phenomenon of drug resistance to conventional therapies due to the abuse and misuse of antibiotics in the agro-food sector has led to the enhanced development of new alternative antibacterial agents. Furthermore, newly implemented green policies are increasingly aimed at combating the use of agrochemicals due to their risk to humans, animals, and the environment. In the last few decades, in fact, extracts from plants rich in flavonoids and phenols have been demonstrated to have powerful antibacterial and antibiofilm activity and other pharmacological actions. *Moringa oleifera* Lam. is marketed as a health supplement primarily for its antibacterial, antioxidant, and anti-inflammatory properties. In the present study, *M. oleifera* was found to inhibit biofilm formation by *X. campestris* pv. *campestris*. As the symptoms observed in the plants are the result of xylem vessel occlusion, unblocking the vessels has recently been investigated. For example, a recent study used a bacteriophage-derived protein that was able to hydrolyze EPS in combination with N-acetylcysteine, leading to the unblocking of xylem in a tree infected with *Xylella fastidiosa*, whereas *X. fastidiosa* was not able to adhere to previously treated plants [68].

From this work, it is possible to confirm that the bacterium can adhere to both abiotic and biotic surfaces, and allow biofilm to be developed on them. In addition, while under the effects of *M. oleifera* extracts, the bacterium changes its cell shape and interrupts the ultrastructural organization that characterizes a developed biofilm, in the adhesion part of the process in particular, and this may have important implications for applications in disease control.

## Figures and Tables

**Table 1:** Effects of biofilm formation on biotic surface. Total viable colony count data are reported as the mean of three different experiments performed in triplicate ± standard deviation.

	Control	MeOH-MOL	HA-MOL	HAMD-MOL
Total Viable Count	2.6 ± 1.44 × 10^8^ CFU/mL	3.03 ± 1.52 × 10^4^ CFU/mL	2.59 ± 2.1 × 10^4^ CFU/mL	6.02 ± 3.65 × 10^3^ CFU/mL

**Table 2:** *X. campestris* pv. *campestris* bacterial isolates.

Bacteria	Sample ID	Host	Province of Isolation	Year of Isolation
*Xanthomonas campestris* pv. *campestris*	10863	Brassica seeds	Bologna	2011
*Xanthomonas campestris* pv. *campestris*	11043	Brassica seeds	Bologna	2011
*Xanthomonas campestris* pv. *campestris*	15616	Brassica seeds	Forlì-Cesena	2011
*Xanthomonas campestris* pv. *campestris*	15619	Brassica seeds	Forlì-Cesena	2012
*Xanthomonas campestris* pv. *campestris*	15622	Brassica seeds	Forlì-Cesena	2012
*Xanthomonas campestris* pv. *campestris*	30788	Brassica seeds	Ravenna	2014
*Xanthomonas campestris* pv. *campestris*	3586	Brassica	DSMZ	1995

## Data Availability

Not applicable.

## Supplementary Materials

The following supporting information can be downloaded at: https://www.mdpi.com/article/10.3390/plants12071508/s1, Table S1: Total phenol and flavonoid content of leaf extracts of M. oleifera Lam.; Table S2: Percentages of chlorogenic acid, rutin, ellagic acid, ferulic acid, and quercetin in the five different dried extracts of M. oleifera Lam. leaves. Each value was obtained from three analyses (mean ± SD); Table S3: Statistical analysis of membrane permeability alteration by single phenols; Table S4: Statistical analysis of single strains differences on biofilm formation by extracts activity.; Table S5: Statistical analysis of single strains differences on biofilm removal by extracts activity; Figure S1: In vitro membrane permeability assay on single strains by single phenols; Figure S2: In vitro biofilm formation assay on single strains by single phenols; Figure S3: In vitro biofilm formation assay on single strains; Figure S4: In vitro biofilm removal assay on single strains.
