# Supplementary material for: Effects of Flavonoids and Phenols from Moringa oleifera Leaf Extracts on Biofilm Processes in Xanthomonas campestris pv. campestris
Source: Plants (Basel). 2023 Mar 30;12(7):1508. doi: 10.3390/plants12071508 (PMC10096499; doi:10.3390/plants12071508)
Supplement: Supplementary file 1 [file plants-12-01508-s001.zip › plants-2110918-supplementary.pdf]

1. *Total Polyphenol Content*

To evaluate the polyphenol content of the extracts was evaluated by the adapted and optimized procedure of Folin–Ciocalteu [1]. Gallic acid was used as a standard to obtain a calibration curve (0–500 ppm). 20 μL of each extract were mixed with Folin- Ciocalteu reagent diluted in water (1.5 mL) and incubated at room temperature for 5 min. Then 300 μL of a sodium carbonate solution was added and then incubated at room temperature in the dark for a further 90 min. Finally, the absorbance is measured by UV- VIS spectrophotometer at 765 nm (UV-31 Scan ONDA) against a blank containing distilled water instead of the extracts. The results were expressed as equivalent to micrograms of gallic acid equivalent (GAE) per milligram of a sample (μg of GAE/mg of dry extract).

2. *Determination of Total Flavonoid Content*

Total flavonoid content was determined following the method by Singh et al. (2012) and Jinting et al. (2017) [2], [3]. Accurately, 1 mL of sample or standard was diluted with 4 mL distilled water and 0.3 mL of 5% sodium nitrate solution was added. After 6 min, 0.3 mL of a 10% aluminum chloride solution was added to the mixture. The mixture was incubated at room temperature for 5 min. Then, 2 mL of 1 M sodium hydroxide was added to the mixture after 5 min of incubation. The mixture was vortexed thoroughly and the absorbance of the yellow color was measured at 510 nm against a blank by using UV-31 Scan ONDA Spectrophotometer. Quercetin was used for the calibration curve with a concentration range of 10–1000 μg/mL. Results were expressed as micrograms quercetin equivalent (μg QE g-1) of dried extract. All experiments were carried out in four replicates.

3. *Characterization of Polyphenols*

3.1 Preparation of Standard Solutions and Sample

Standard solutions of chlorogenic acid, rutin, ellagic acid, ferulic acid and quercetin were prepared and diluted in methanol to obtain the final concentration in the range 0.625–80 μg/mL. A carefully weighed aliquot (20 mg) of each extract was dissolved in methanol or methanol-water. Each solution was filtered through a 0.45 nylon membrane filter and subsequently analyzed in triplicate by HPLC.

3.2 HPLC Apparatus and Chromatographic Conditions

HPLC analysis was performed using an Agilent 1100 Series HPLC System equipped with a G1315A DAD and with a Hydro RP18 Sinergi 80A column (4.6 × 250 mm, 4 μm) from Phenomenex. Separation was monitored with absorbance detection at a wavelength of 254 ± 8 nm. The elution was performed on a gradient solvent using solvent A (water 0.01 M H_3PO_4) and solvent B (acetonitrile 0.01 M H_3PO_4). The ratios were as follows: 90:10 (A/B) to 80:20 (A/B) in 5 min, held for 5 min, 80:20 (A/B) to 20:80 (A/B) in 10 min, 20:80 (A/B) to 90:10 (A/B) in 2 min. The flow rate was 1.2 mL/min at room temperature. The injection volume for all samples and standards was 5 μL. The quantitative HPLC analysis was calculated, for each compound, according to its peak area.

4. *Quantitative and Qualitative Estimations of Polyphenols*

For the current study, five different extracts of Moringa oleifera leaves were prepared and analyzed: hydroalcoholic, methanolic, infusion, hydroalcoholic extract with maltodex- trins, and water extract with maltodextrins. The analyzes carried out in this study, in particular the Folin–Ciocalteu test and the total flavonoid content test, allowed to highlight a good content of polyphenols in all the samples tested (Table S1). It was therefore decided to carry out a characterization by HPLC which allowed to identify and quantify some of the polyphenols present. In particular, the analyses focused on active ingredients such as chlorogenic acid, ellagic acid, ferulic acid, rutin and quercetin, polyphenols of interest for their antimicrobial activity [4]. The

presence of these phenols had already been previously highlighted in M. oleifera Lam. leaf extracts from Senegal [5]. Overall (Table S2) the most present active ingredients were found to be ferulic acid, rutin and also chlorogenic acid, except for the WMD-MOE extract that showed the lowest values compared to all the other samples. Specifically, HA-MOE was certainly the one that showed a higher number of polyphenols, presenting an excellent percentage of rutin and ferulic acid. HAMD-MOE and MeOH-MOE follow in terms of percentages of polyphenols: the first showed a good presence of chlorogenic acid, ellagic acid and ferulic acid, while the methanolic extract showed a peak of rutin and ferulic acid. In-MOE and WMD-MOE both reveal the lowest polyphenolic profile of all the samples analyzed, the significant percentages of active ingredients were ferulic acid and chlorogenic acid, respectively. The only active ingredient that was not identified in any of the analyzed extracts is quercetin.

Table S1. Total phenol and flavonoid content of leaf extracts of M. oleifera Lam.

	Total Phenol Content (μg GAE/mg)	Total Flavonoid Content (μg QE/mg)
HA-MOE	45.63 ± 3.41	601.25 ± 44.00
MeOH-MOE	36.37 ± 2.33	1611.98 ± 70.44
In-MOE	37.56 ± 2.77	193.88 ± 1.74
WMD-MOE	25.65 ± 1.20	176.67 ± 9.56
HAMD-MOE	42.49 ± 1.39	291.07 ± 14.91

Table S2. Percentages of chlorogenic acid, rutin, ellagic acid, ferulic acid, and quercetin in the five different dried extracts of M. oleifera Lam. leaves. Each value was obtained from three analyses (mean ± SD).

	Percentage of Detected Compounds (*w*/*w*)				
	Chlorogenic Acid	Rutin	Ellagic Acid	Ferulic Acid	Quercetin
HA-MOE	0.50 ± 0.04	1.05 ± 0.04	0.20 ± 0.01	2.22 ± 0.03	-[1]
MeOH-MOE	0.34 ± 0.02	0.84 ± 0.04	0.20 ± 0.01	1.12 ± 0.11	-[1]
In-MOE	0.29 ± 0.03	0.49 ± 0.01	0.06 ± 0.00	1.17 ± 0.03	-[1]
WMD-MOE	0.43 ± 0.02	0.30 ± 0.02	0.10 ± 0.00	0.35 ± 0.01	-[1]
HAMD-MOE	0.90 ± 0.04	0.63 ± 0.034	0.21 ± 0.01	0.91 ± 0.00	-[1]

5. *Additional statistical analysis, graphs and tables*

Table S3. Statistical analysis of membrane permeability alteration by single phenols.

Tukey's multiple comparisons test	*Mean Diff,*	*95,00% CI of diff,*	*Significant?*	*Summary*	*Adjusted P Value*
Control (Untreated Xcc) vs. HAMD-MOE (Positive control)	-78,67	-87,02 to -70,31	Yes	****	<0,0001
Control (Untreated Xcc) vs. Rutin	-53,67	-62,02 to -45,31	Yes	****	<0,0001
Control (Untreated Xcc) vs. Quercetin	-20,33	-28,69 to -11,98	Yes	****	<0,0001
Control (Untreated Xcc) vs. Chlorogenic Acid	-59,33	-67,69 to -50,98	Yes	****	<0,0001
Control (Untreated Xcc) vs. Ellagic Acid	-50,67	-59,02 to -42,31	Yes	****	<0,0001
HAMD-MOE (Positive control) vs. Rutin	25	16,65 to 33,35	Yes	****	<0,0001
HAMD-MOE (Positive control) vs. Quercetin	58,33	49,98 to 66,69	Yes	****	<0,0001
HAMD-MOE (Positive control) vs. Chlorogenic Acid	19,33	10,98 to 27,69	Yes	****	<0,0001
HAMD-MOE (Positive control) vs. Ellagic Acid	28	19,65 to 36,35	Yes	****	<0,0001
Rutin vs. Quercetin	33,33	24,98 to 41,69	Yes	****	<0,0001
Rutin vs. Chlorogenic Acid	-5,667	-14,02 to 2,687	No	ns	0,2735
Rutin vs. Ellagic Acid	3	-5,354 to 11,35	No	ns	0,8259
Quercetin vs. Chlorogenic Acid	-39	-47,35 to -30,65	Yes	****	<0,0001
Quercetin vs. Ellagic Acid	-30,33	-38,69 to -21,98	Yes	****	<0,0001
Chlorogenic Acid vs. Ellagic Acid	8,667	0,3130 to 17,02	Yes	*	0,0405

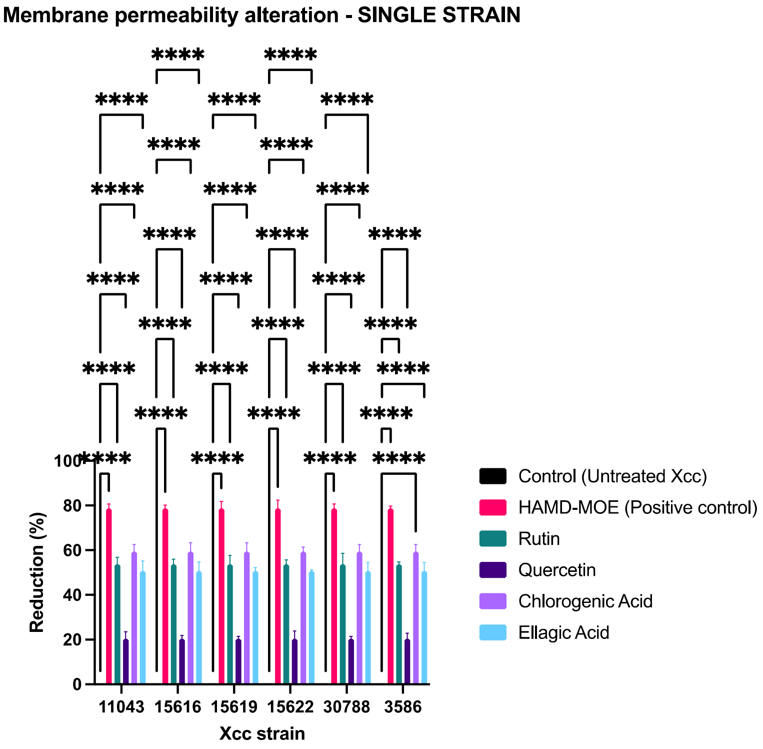

Figure S1. *In vitro* membrane permeability assay. *X. campestris* pv. *campestris* permeability was assessed by PI intake compared to untreated control. Data represent average of three independent experiments on three copies (mean +/- standard deviation), and values are given as percentages; **** p < 0.001.

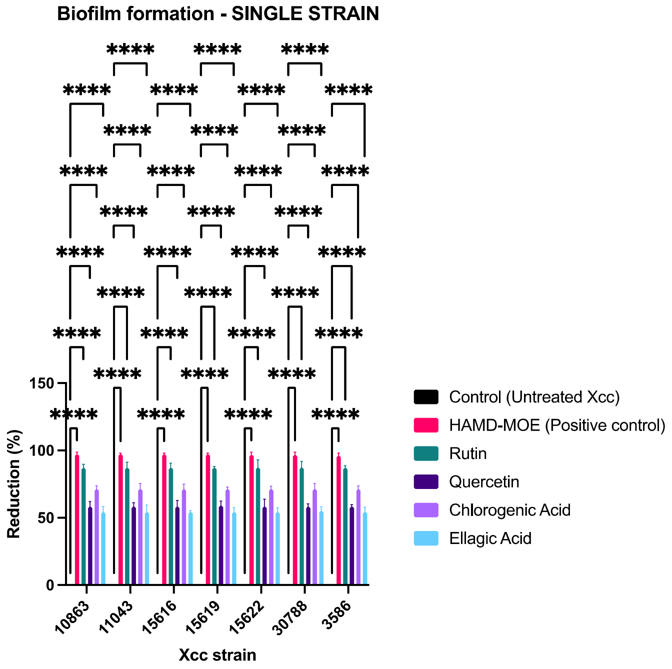

Figure S2. *In vitro* biofilm formation assay. *X. campestris* pv. *campestris* biofilm formation was assessed with crystal violet method, presented as percentage compared to untreated control. Data represent average of three independent experiments on three copies (mean +/- standard deviation), and values are given as percentages; **** $p < 0.001$

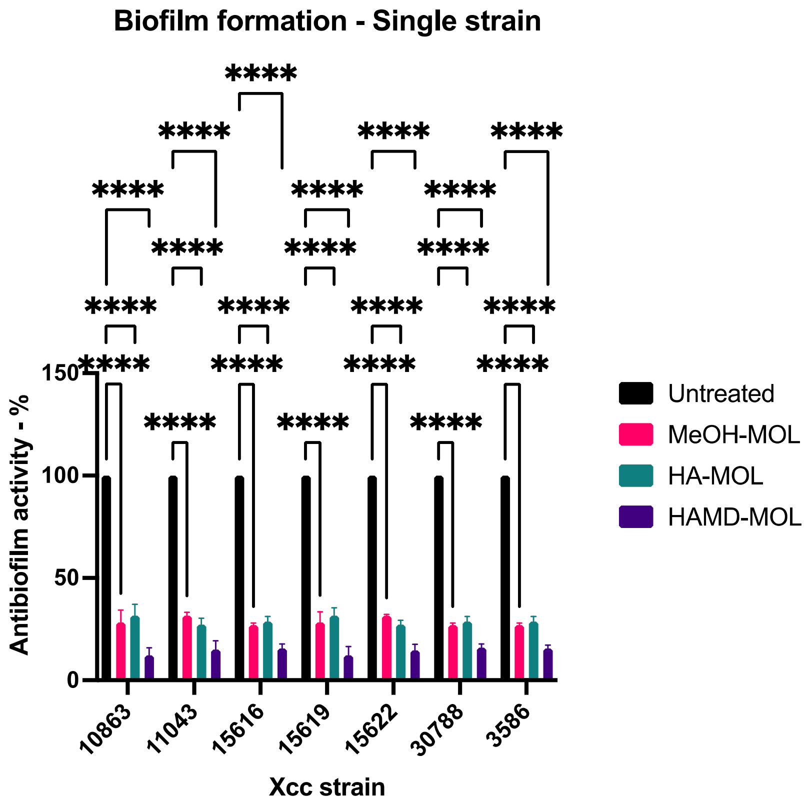

Figure S3. *In vitro* biofilm formation assay. *X. campestris* pv. *campestris* biofilm was measured by OD600, presented as percentage compared to untreated control. Data represent average of three independent experiments on three copies (mean +/- standard deviation), and values are given as percentages; **** $p < 0.001$.

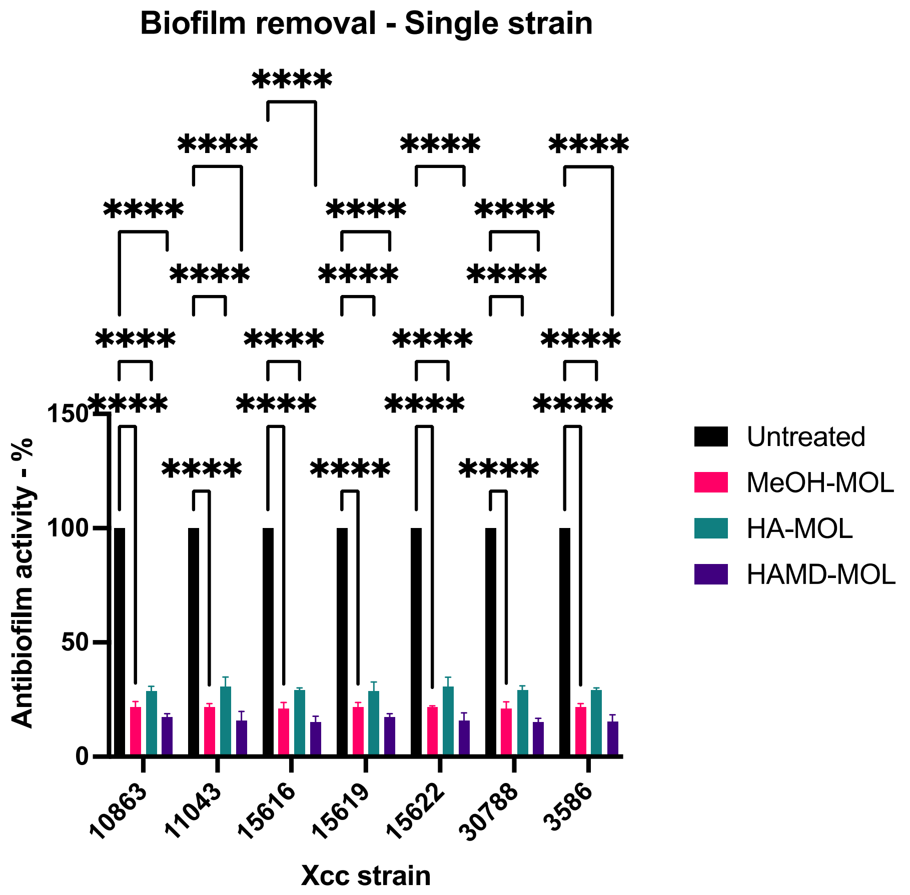

Figure S4. *In vitro* biofilm removal assay. *X. campestris* pv. *campestris* biofilm was measured by OD600, presented as percentage compared to untreated control. Data represent average of three independent experiments on three copies (mean +/- standard deviation), and values are given as percentages; **** p < 0.001.

Table S4. Statistical analysis of single strains differences on biofilm formation by extracts activity.

Tukey's multiple comparisons test	*Mean Diff,*	*95,00% CI of diff,*	*Summary*	*Adjusted P Value*
Untreated				
10863 vs. 11043	0,000	-6,892 to 6,892	ns	>0,9999
10863 vs. 15616	0,000	-6,892 to 6,892	ns	>0,9999
10863 vs. 15619	0,000	-6,892 to 6,892	ns	>0,9999
10863 vs. 15622	0,000	-6,892 to 6,892	ns	>0,9999
10863 vs. 30788	0,000	-6,892 to 6,892	ns	>0,9999
10863 vs. 3586	0,000	-6,892 to 6,892	ns	>0,9999
11043 vs. 15616	0,000	-6,892 to 6,892	ns	>0,9999
11043 vs. 15619	0,000	-6,892 to 6,892	ns	>0,9999
11043 vs. 15622	0,000	-6,892 to 6,892	ns	>0,9999
11043 vs. 30788	0,000	-6,892 to 6,892	ns	>0,9999
11043 vs. 3586	0,000	-6,892 to 6,892	ns	>0,9999
15616 vs. 15619	0,000	-6,892 to 6,892	ns	>0,9999
15616 vs. 15622	0,000	-6,892 to 6,892	ns	>0,9999
15616 vs. 30788	0,000	-6,892 to 6,892	ns	>0,9999
15616 vs. 3586	0,000	-6,892 to 6,892	ns	>0,9999
15619 vs. 15622	0,000	-6,892 to 6,892	ns	>0,9999
15619 vs. 30788	0,000	-6,892 to 6,892	ns	>0,9999
15619 vs. 3586	0,000	-6,892 to 6,892	ns	>0,9999
15622 vs. 30788	0,000	-6,892 to 6,892	ns	>0,9999

15622 vs. 3586	0,000	-6,892 to 6,892	ns	>0,9999
30788 vs. 3586	0,000	-6,892 to 6,892	ns	>0,9999
**MeOH-MOL**				
10863 vs. 11043	-3,333	-10,23 to 3,559	ns	0,7558
10863 vs. 15616	1,333	-5,559 to 8,225	ns	0,9968
10863 vs. 15619	0,000	-6,892 to 6,892	ns	>0,9999
10863 vs. 15622	-3,333	-10,23 to 3,559	ns	0,7558
10863 vs. 30788	1,333	-5,559 to 8,225	ns	0,9968
10863 vs. 3586	1,333	-5,559 to 8,225	ns	0,9968
11043 vs. 15616	4,667	-2,225 to 11,56	ns	0,3839
11043 vs. 15619	3,333	-3,559 to 10,23	ns	0,7558
11043 vs. 15622	0,000	-6,892 to 6,892	ns	>0,9999
11043 vs. 30788	4,667	-2,225 to 11,56	ns	0,3839
11043 vs. 3586	4,667	-2,225 to 11,56	ns	0,3839
15616 vs. 15619	-1,333	-8,225 to 5,559	ns	0,9968
15616 vs. 15622	-4,667	-11,56 to 2,225	ns	0,3839
15616 vs. 30788	0,000	-6,892 to 6,892	ns	>0,9999
15616 vs. 3586	0,000	-6,892 to 6,892	ns	>0,9999
15619 vs. 15622	-3,333	-10,23 to 3,559	ns	0,7558
15619 vs. 30788	1,333	-5,559 to 8,225	ns	0,9968
15619 vs. 3586	1,333	-5,559 to 8,225	ns	0,9968
15622 vs. 30788	4,667	-2,225 to 11,56	ns	0,3839
15622 vs. 3586	4,667	-2,225 to 11,56	ns	0,3839
30788 vs. 3586	0,000	-6,892 to 6,892	ns	>0,9999
**HA-MOL**				
10863 vs. 11043	4,333	-2,559 to 11,23	ns	0,4744
10863 vs. 15616	3,000	-3,892 to 9,892	ns	0,8344
10863 vs. 15619	0,000	-6,892 to 6,892	ns	>0,9999
10863 vs. 15622	4,333	-2,559 to 11,23	ns	0,4744
10863 vs. 30788	3,000	-3,892 to 9,892	ns	0,8344
10863 vs. 3586	3,000	-3,892 to 9,892	ns	0,8344
11043 vs. 15616	-1,333	-8,225 to 5,559	ns	0,9968
11043 vs. 15619	-4,333	-11,23 to 2,559	ns	0,4744
11043 vs. 15622	0,000	-6,892 to 6,892	ns	>0,9999
11043 vs. 30788	-1,333	-8,225 to 5,559	ns	0,9968
11043 vs. 3586	-1,333	-8,225 to 5,559	ns	0,9968
15616 vs. 15619	-3,000	-9,892 to 3,892	ns	0,8344
15616 vs. 15622	1,333	-5,559 to 8,225	ns	0,9968
15616 vs. 30788	0,000	-6,892 to 6,892	ns	>0,9999
15616 vs. 3586	0,000	-6,892 to 6,892	ns	>0,9999
15619 vs. 15622	4,333	-2,559 to 11,23	ns	0,4744
15619 vs. 30788	3,000	-3,892 to 9,892	ns	0,8344
15619 vs. 3586	3,000	-3,892 to 9,892	ns	0,8344
15622 vs. 30788	-1,333	-8,225 to 5,559	ns	0,9968
15622 vs. 3586	-1,333	-8,225 to 5,559	ns	0,9968
30788 vs. 3586	0,000	-6,892 to 6,892	ns	>0,9999
**HAMD-MOL**				
10863 vs. 11043	-2,667	-9,559 to 4,225	ns	0,8975
10863 vs. 15616	-3,333	-10,23 to 3,559	ns	0,7558
10863 vs. 15619	0,000	-6,892 to 6,892	ns	>0,9999
10863 vs. 15622	-2,333	-9,225 to 4,559	ns	0,9435
10863 vs. 30788	-3,667	-10,56 to 3,225	ns	0,6658
10863 vs. 3586	-3,333	-10,23 to 3,559	ns	0,7558
11043 vs. 15616	-0,6667	-7,559 to 6,225	ns	>0,9999
11043 vs. 15619	2,667	-4,225 to 9,559	ns	0,8975
11043 vs. 15622	0,3333	-6,559 to 7,225	ns	>0,9999
11043 vs. 30788	-1,000	-7,892 to 5,892	ns	0,9994
11043 vs. 3586	-0,6667	-7,559 to 6,225	ns	>0,9999

15616 vs. 15619	3,333	-3,559 to 10,23	ns	0,7558
15616 vs. 15622	1,000	-5,892 to 7,892	ns	0,9994
15616 vs. 30788	-0,3333	-7,225 to 6,559	ns	>0,9999
15616 vs. 3586	0,000	-6,892 to 6,892	ns	>0,9999
15619 vs. 15622	-2,333	-9,225 to 4,559	ns	0,9435
15619 vs. 30788	-3,667	-10,56 to 3,225	ns	0,6658
15619 vs. 3586	-3,333	-10,23 to 3,559	ns	0,7558
15622 vs. 30788	-1,333	-8,225 to 5,559	ns	0,9968
15622 vs. 3586	-1,000	-7,892 to 5,892	ns	0,9994
30788 vs. 3586	0,3333	-6,559 to 7,225	ns	>0,9999

Table S5. Statistical analysis of single strains differences on biofilm removal by extracts activity.

Tukey's multiple comparisons test	*Mean Diff,*	*95,00% CI of diff,*	*Summary*	*Adjusted P Value*
Untreated				
10863 vs. 11043	0,000	-5,675 to 5,675	ns	>0,9999
10863 vs. 15616	0,000	-5,675 to 5,675	ns	>0,9999
10863 vs. 15619	0,000	-5,675 to 5,675	ns	>0,9999
10863 vs. 15622	0,000	-5,675 to 5,675	ns	>0,9999
10863 vs. 30788	0,000	-5,675 to 5,675	ns	>0,9999
10863 vs. 3586	0,000	-5,675 to 5,675	ns	>0,9999
11043 vs. 15616	0,000	-5,675 to 5,675	ns	>0,9999
11043 vs. 15619	0,000	-5,675 to 5,675	ns	>0,9999
11043 vs. 15622	0,000	-5,675 to 5,675	ns	>0,9999
11043 vs. 30788	0,000	-5,675 to 5,675	ns	>0,9999
11043 vs. 3586	0,000	-5,675 to 5,675	ns	>0,9999
15616 vs. 15619	0,000	-5,675 to 5,675	ns	>0,9999
15616 vs. 15622	0,000	-5,675 to 5,675	ns	>0,9999
15616 vs. 30788	0,000	-5,675 to 5,675	ns	>0,9999
15616 vs. 3586	0,000	-5,675 to 5,675	ns	>0,9999
15619 vs. 15622	0,000	-5,675 to 5,675	ns	>0,9999
15619 vs. 30788	0,000	-5,675 to 5,675	ns	>0,9999
15619 vs. 3586	0,000	-5,675 to 5,675	ns	>0,9999
15622 vs. 30788	0,000	-5,675 to 5,675	ns	>0,9999
15622 vs. 3586	0,000	-5,675 to 5,675	ns	>0,9999
30788 vs. 3586	0,000	-5,675 to 5,675	ns	>0,9999
MeOH-MOL				
10863 vs. 11043	0,000	-5,675 to 5,675	ns	>0,9999
10863 vs. 15616	0,6667	-5,009 to 6,342	ns	0,9998
10863 vs. 15619	0,000	-5,675 to 5,675	ns	>0,9999
10863 vs. 15622	0,000	-5,675 to 5,675	ns	>0,9999
10863 vs. 30788	0,6667	-5,009 to 6,342	ns	0,9998
10863 vs. 3586	0,000	-5,675 to 5,675	ns	>0,9999
11043 vs. 15616	0,6667	-5,009 to 6,342	ns	0,9998
11043 vs. 15619	0,000	-5,675 to 5,675	ns	>0,9999
11043 vs. 15622	0,000	-5,675 to 5,675	ns	>0,9999
11043 vs. 30788	0,6667	-5,009 to 6,342	ns	0,9998
11043 vs. 3586	0,000	-5,675 to 5,675	ns	>0,9999
15616 vs. 15619	-0,6667	-6,342 to 5,009	ns	0,9998
15616 vs. 15622	-0,6667	-6,342 to 5,009	ns	0,9998
15616 vs. 30788	0,000	-5,675 to 5,675	ns	>0,9999
15616 vs. 3586	-0,6667	-6,342 to 5,009	ns	0,9998
15619 vs. 15622	0,000	-5,675 to 5,675	ns	>0,9999
15619 vs. 30788	0,6667	-5,009 to 6,342	ns	0,9998
15619 vs. 3586	0,000	-5,675 to 5,675	ns	>0,9999
15622 vs. 30788	0,6667	-5,009 to 6,342	ns	0,9998
15622 vs. 3586	0,000	-5,675 to 5,675	ns	>0,9999
30788 vs. 3586	-0,6667	-6,342 to 5,009	ns	0,9998

HA-MOL				
10863 vs. 11043	-2,000	-7,675 to 3,675	ns	0,9321
10863 vs. 15616	-0,3333	-6,009 to 5,342	ns	>0,9999
10863 vs. 15619	0,000	-5,675 to 5,675	ns	>0,9999
10863 vs. 15622	-2,000	-7,675 to 3,675	ns	0,9321
10863 vs. 30788	-0,3333	-6,009 to 5,342	ns	>0,9999
10863 vs. 3586	-0,3333	-6,009 to 5,342	ns	>0,9999
11043 vs. 15616	1,667	-4,009 to 7,342	ns	0,9714
11043 vs. 15619	2,000	-3,675 to 7,675	ns	0,9321
11043 vs. 15622	0,000	-5,675 to 5,675	ns	>0,9999
11043 vs. 30788	1,667	-4,009 to 7,342	ns	0,9714
11043 vs. 3586	1,667	-4,009 to 7,342	ns	0,9714
15616 vs. 15619	0,3333	-5,342 to 6,009	ns	>0,9999
15616 vs. 15622	-1,667	-7,342 to 4,009	ns	0,9714
15616 vs. 30788	0,000	-5,675 to 5,675	ns	>0,9999
15616 vs. 3586	0,000	-5,675 to 5,675	ns	>0,9999
15619 vs. 15622	-2,000	-7,675 to 3,675	ns	0,9321
15619 vs. 30788	-0,3333	-6,009 to 5,342	ns	>0,9999
15619 vs. 3586	-0,3333	-6,009 to 5,342	ns	>0,9999
15622 vs. 30788	1,667	-4,009 to 7,342	ns	0,9714
15622 vs. 3586	1,667	-4,009 to 7,342	ns	0,9714
30788 vs. 3586	0,000	-5,675 to 5,675	ns	>0,9999
HAMD-MOL				
10863 vs. 11043	1,667	-4,009 to 7,342	ns	0,9714
10863 vs. 15616	2,333	-3,342 to 8,009	ns	0,8680
10863 vs. 15619	0,000	-5,675 to 5,675	ns	>0,9999
10863 vs. 15622	1,667	-4,009 to 7,342	ns	0,9714
10863 vs. 30788	2,333	-3,342 to 8,009	ns	0,8680
10863 vs. 3586	2,000	-3,675 to 7,675	ns	0,9321
11043 vs. 15616	0,6667	-5,009 to 6,342	ns	0,9998
11043 vs. 15619	-1,667	-7,342 to 4,009	ns	0,9714
11043 vs. 15622	0,000	-5,675 to 5,675	ns	>0,9999
11043 vs. 30788	0,6667	-5,009 to 6,342	ns	0,9998
11043 vs. 3586	0,3333	-5,342 to 6,009	ns	>0,9999
15616 vs. 15619	-2,333	-8,009 to 3,342	ns	0,8680
15616 vs. 15622	-0,6667	-6,342 to 5,009	ns	0,9998
15616 vs. 30788	0,000	-5,675 to 5,675	ns	>0,9999
15616 vs. 3586	-0,3333	-6,009 to 5,342	ns	>0,9999
15619 vs. 15622	1,667	-4,009 to 7,342	ns	0,9714
15619 vs. 30788	2,333	-3,342 to 8,009	ns	0,8680
15619 vs. 3586	2,000	-3,675 to 7,675	ns	0,9321
15622 vs. 30788	0,6667	-5,009 to 6,342	ns	0,9998
15622 vs. 3586	0,3333	-5,342 to 6,009	ns	>0,9999
30788 vs. 3586	-0,3333	-6,009 to 5,342	ns	>0,9999

[1] V. L. Singleton and J. A. Rossi, “Colorimetry of Total Phenolics with Phosphomolybdic-Phosphotungstic Acid Reagents,” *Am J Enol Vitic*, vol. 16, no. 3, p. 144, Jan. 1965, [Online]. Available: http://www.ajevonline.org/content/16/3/144.abstract
[2] V. Singh, N. Guizani, M. E. Musthafa, and L. Hakkim, “Comparative analysis of total phenolics, flavonoid content and antioxidant profile of different date varieties (Phoenix dactylifera L.) From Sultanate of Oman,” 2012. [Online]. Available: https://www.researchgate.net/publication/277309552
[3] J. Azieana, M. N. Zainon, A. Noriham, and M. N. Rohana, “Total Phenolic and Flavonoid Content and Antioxidant Activities of Ten Malaysian Wild Mushrooms,” *OAlib*, vol. 04, no. 11, pp. 1–9, 2017, doi: 10.4236/oalib.1103987.
[4] W. Nouman, F. Anwar, T. Gull, A. Newton, E. Rosa, and R. Domínguez-Perles, “Profiling of polyphenolics, nutrients and antioxidant potential of germplasm’s leaves from seven cultivars of Moringa oleifera Lam.,” *Ind Crops Prod*, vol. 83, pp. 166–176, May 2016, doi: 10.1016/j.indcrop.2015.12.032.
[5] A. Baldisserotto *et al.*, “Moringa oleifera leaf extracts as multifunctional ingredients for ‘natural and organic’ sunscreens and photoprotective preparations,” *Molecules*, vol. 23, no. 3, 2018, doi: 10.3390/molecules23030664.
